# Do all adolescents benefit equally from school-based mental health interventions? A subgroup analysis by depression severity at 12 and 18 months follow-up

**DOI:** 10.3389/fpsyt.2026.1826587

**Published:** 2026-08-10

**Authors:** Denis Duagi, Ben Carter, Steven Lisk, June S.L. Brown

**Affiliations:** Institute of Psychiatry, Psychology and Neuroscience, King’s College London, London, United Kingdom

**Keywords:** anxiety, CBT, depression, school-based interventions, youth mental health

## Abstract

**Background:**

School-based mental health interventions show modest effects in reducing depression symptoms. However, most trials report only average effects that may mask important differences across symptom severity levels, which are important in assessing long term risk, particularly as sub-threshold depression is a risk factor for clinical depression.

**Aims:**

This study examined whether the effects of DISCOVER, a brief CBT-based self-referral workshop for adolescents, at 12- and 18-month follow-ups differed according to participants’ baseline depression severity (clinical, subthreshold, or normal range).

**Methods:**

This is an exploratory secondary analysis of the BESST cluster randomised controlled trial involving 900 adolescents from 57 UK schools randomised to DISCOVER or treatment-as-usual (TAU). Participants were categorised by baseline depression severity using established Mood and Feelings Questionnaire (MFQ) cut-offs: clinical (>27), subthreshold (17-27), or normal (<17). We conducted multilevel mixed-effects multinomial logistic regression to examine transitions between severity categories and mixed-effects linear models for continuous outcomes (depression, anxiety, wellbeing, sleep quality, resilience) for each severity group.

**Results:**

Among adolescents with clinical-level depression at baseline (DISCOVER n=142, TAU n=172), DISCOVER significantly increased the odds of achieving normal-range symptoms at 12 months (RRR = 3.12, 95% CI: 1.48-15.97, p=0.006) and 18 months (RRR = 2.63, 95% CI: 1.11-6.24, p=0.029). Small-to-moderate effects were maintained for depression (d=0.44 at 12 months, d=0.30 at 18 months) and anxiety (d=0.38 at 12 months, d=0.30 at 18 months). For adolescents with subthreshold depression symptoms at baseline (DISCOVER n=170, TAU n=157), DISCOVER did not show benefits on depression but demonstrated specific anxiety benefits at 12 months (d=0.34, p=0.004).

**Conclusions:**

DISCOVER produces small to moderate sustained benefits for adolescents with clinical-level depression, with effects maintained through critical post-school transitions. The intervention shows more limited benefits for those with subthreshold symptoms, achieving specific anxiety reduction but not broader improvement. These findings suggest that self-referral school-based interventions are effective over the longer term for adolescents with clinical-level symptoms, while highlighting the need for tailored approaches for subthreshold presentations.

## 1 Introduction

Schools represent an optimal platform for delivering early mental health interventions given their established roles in adolescents’ daily lives, extensive reach, and capacity to bypass traditional healthcare access constraints (1). Recent initiatives have embraced school settings as essential channels for enhancing adolescents’ mental health support, for example, through the introduction of specialised education mental health support teams in schools (2). Nevertheless, the evidence of effectiveness remains complex and varied, with systematic reviews documenting moderate effects for reducing anxiety yet inconsistent outcomes for depression (3, 4). This mixed evidence underscores the necessity of examining which intervention characteristics determine effectiveness for different subgroups of adolescents.

A critical yet under-examined question concerns adolescents with subthreshold depressive symptoms—those who experience clinically significant distress without meeting full diagnostic criteria. Despite affecting a substantial proportion of UK adolescents (5, 6), this group receives limited attention in intervention research, even though they display functional impairments comparable to peers with clinical depression and face elevated risk for future psychopathology (7–9). Early intervention targeting subthreshold symptoms may offer protective effects by preventing progression to clinical depression, yet most school-based intervention trials either exclude these adolescents through targeted screening or obscure their outcomes within universal program averages (10). Understanding whether interventions benefit adolescents across the spectrum of depressive symptoms—from minimal to clinical—is therefore essential for developing effective preventive strategies.

Current approaches to implementing school-based mental health interventions predominantly adopt either targeted or universal strategies, each with distinct limitations for reaching adolescents with subthreshold symptoms. Targeted approaches selectively intervene with students identified through screenings as exhibiting symptoms, generally demonstrating greater efficacy but raising substantial ethical concerns including potential stigmatization and inadvertent exclusion of help-avoidant students (11–13). Conversely, universal interventions aim to reach all students, theoretically minimizing stigma, but frequently show diluted effects for those with existing symptoms and limited efficacy when delivered by educators rather than specialists (14, 15). Critically, average effects across entire populations may mask differential responses among subgroups; recent evidence suggests universal interventions may even worsen outcomes for students with pre-existing difficulties (16), and meta-analyses increasingly indicate they are not effective overall (17).

An innovative alternative is the self-referral approach, enabling adolescents to decide upon involvement based on personal recognition of need rather than external diagnosis. Originally developed for adults (18), community self-referral CBT stress workshops effectively engaged participants who had not previously sought professional assistance, including significant proportions with diagnosable mental health problems (19, 20). The adolescent DISCOVER workshops successfully replicated these engagement findings, enrolling previously untreated students, including those from ethnic minority groups (21).

Importantly, the self-referral mechanism attracted students across the full spectrum of depressive symptoms, creating a unique opportunity to examine whether a single intervention can benefit adolescents at different severity levels and whether it offers protective effects for those with minimal or subthreshold symptoms at baseline.

This study investigates differential treatment effects within the BESST trial by examining 12- and 18-month outcomes across subgroups defined by baseline depression severity. Specifically, it addresses two key questions: First, do adolescents with subthreshold depression benefit from the intervention to the same degree as those with clinical-level symptoms? Second, is the intervention associated with patterns consistent with potential prevention of symptom escalation?

## 2 Materials and methods

### 2.1 Study design

The current study reports the long-term outcomes at 12 and 18 months from the Brief Educational Workshops in Secondary Schools Trial (BESST), a two-arm, single-blind, multi-centre cluster randomised controlled trial (cRCT). The trial compared the DISCOVER intervention, a school-based psychological stress management workshop programme, with treatment-as-usual (TAU), representing standard school-based mental health support.

A detailed protocol of the BESST Trial was published elsewhere (22), however, information about the study design is provided below:

#### 2.1.1 Clinicians

A new professional group called Mental Health in Schools Team (MHST) who were working with schools as part of CAMHS clinical services were recruited across four regions in the UK: London (Bexley, Enfield, Greenwich, Hackney, Islington, Newham), Midlands (Burton, Shrewsbury, Solihull), Northwest (Cheshire, Liverpool, Manchester) and Southwest (Bath and Northeast Somerset, Bristol, Wiltshire).

#### 2.1.2 Schools

Schools were recruited according to eligibility criteria and willingness to work alongside the research team to organise the implementation of the trial. To be eligible, the schools needed to be state-funded, have a minimum of 70 registered sixth form students and have physical space availability to host the trial.

#### 2.1.3 Participants

Participating students needed to be: 1) aged 16–18 years old; 2) seeking psychological help for mild and moderate stress, anxiety, and low mood problems; 3) attending a participating school; 4) fluent in English and 5) able to provide informed written consent to participate. Participants were excluded if they were: 1) identified as actively suicidal through risk assessment, 2) receiving in psychological therapy for anxiety or depression within CAMHS, or 3) had severe learning difficulties.

### 2.2 Ethics

Ethics approval for the BESST Trial was obtained from King’s College London PNM Research Ethics Subcommittee (HR-20/21-17758). An amendment to extend data collection to 12 and 18 months was accepted on 30^th^ March 2022.

All participants were required to give written informed consent; parental consent was not required as participants were above 16 years old. This study is congruent with the General Data Protection Regulation legislation.

### 2.3 Sample size

The BESST trial is powered to 90% and accounts for a dropout rate of 15% based on the feasibility study. The inter-class correlation coefficient (ICC) was 0.03, and power calculations were carried out with an effect size of 0.28 for depression symptoms with alpha set at 0.05. The original sample size calculation indicated that the number of participants needed was 900, from 60 schools. Before the start of the long-term follow-up, 900 participants had already been recruited into the trial, and for the 12-month and 18-month follow-ups, power was reduced to 80% due to dropout rates higher than 25%.

Attrition at follow-up was moderate (25.6% at 12 months; 27.9% at 18 months) and was slightly higher in the DISCOVER arm compared to TAU at both timepoints. Baseline demographic and clinical characteristics of follow-up completers were broadly comparable to the original trial sample, suggesting limited evidence of systematic attrition bias. Given the relatively balanced attrition across groups and the absence of strong, consistent predictors of missingness beyond randomisation status, the missing data mechanism was assumed to be missing at random (MAR). Analyses were conducted using mixed-effects models, which use all available data under the MAR assumption and are considered robust to incomplete outcome data.

### 2.4 Measures

The primary outcome measure was symptoms of depression, measured using the Mood and Feelings Questionnaire (MFQ) (23), a widely-used self-report instrument designed specifically for assessing depressive symptoms in adolescents aged 8–18 years. The MFQ comprises 33 items, each rated on a 3-point Likert scale from 0 (“not true”) to 2 (“true”), resulting in a total score ranging from 0 to 66, with higher scores indicating more severe depressive symptoms. The MFQ has demonstrated strong psychometric properties, including high internal consistency (α = .90–.92), strong criterion validity, and good discriminative capacity for clinical depression among adolescents. Scores above 27 are considered clinically significant, indicating possible depression that warrants further evaluation.

Secondary outcomes and their measures were anxiety, assessed using the anxiety sub-scale from the Revised Child Anxiety and Depression Scale (RCADS; higher scores indicate greater severity of anxiety symptoms)–child version; wellbeing, assessed using the Warwick– Edinburgh Mental Wellbeing Scale (WEWBS; higher scores indicate higher levels of general wellbeing); sleep quality, assessed using the Sleep Condition Indicator (SCI; higher scores indicate better sleep); and resilience, assessed using the Child and Youth Resilience Measure 12 (CYRM-12; higher scores indicate greater resilience).

### 2.5 Participant classification into severity groups

BESST Trial participants were categorised into three severity groups at baseline (clinical, subthreshold, or normal) based on the primary outcome, the Mood and Feelings Questionnaire (MFQ). Those scoring over 27 were considered above the threshold. Participants scoring between 17 and 27 on the MFQ were defined as having subthreshold depression symptoms. The cut-off point of 17 for subthreshold depression was chosen based on a previous study which defined young people with MFQ scores ≥17 as having sub-syndromal depression and being at high risk of clinical depression (24), following receiver operating characteristic (ROC) curve analysis on MFQ data compared with the Schedule for Affective Disorders and Schizophrenia – Child Version (K-SADS) interviews as the gold standard for depressive disorder diagnoses (25).

Participants with MFQ scores >27 were considered to have clinical-level depressive symptoms. This cut-off point was based on two previous studies, which performed ROC curve analysis comparing MFQ scores to diagnoses made using the K-SADS (26) and the Children’s Depression Rating Scale–Revised (CDRS-R) (27).

### 2.6 Interventions

Participants in intervention schools received the DISCOVER programme, a one-day intensive cognitive behavioural therapy (CBT)-based workshop designed specifically for older adolescents aged 16–18, adapted from a community-based psychoeducational model originally developed for adults. Workshops were delivered by a master’s or diploma-level therapist and two assistants (Educational Mental Health Practitioners or Children’s Wellbeing Practitioners), supported by a workbook and individualised goal-setting sessions before and after the workshop.

Workshop content included psychoeducation about stress, interactive role-plays, video presentations, and individual goal-setting to reinforce personal coping skills. Each participant received up to three follow-up telephone calls to review goals, facilitate skill consolidation, and address barriers.

Participants in the TAU group received standard school-based mental health support and were provided with signposting information to local and online mental health resources. TAU across participating schools comprised a broad range of universal and targeted mental health provision, including curriculum-based psychoeducation (e.g., PSHE), peer support, stigma reduction activities, and access to individual-level interventions such as counselling, CBT, and targeted support groups (**Supplementary Table 1**).

### 2.7 Statistical analysis

#### 2.7.1 Likelihood of change in symptom severity

To examine the effect of the intervention on symptom severity at follow-up, we conducted multilevel mixed-effects multinomial logistic regression analyses with depression severity at 12- and 18-months (categorised as clinical, subthreshold, or normal) as the outcome variable, using the meqrlogit command in STATA (Version 17). A random intercept was set for school, and the outcome was modelled with clinical severity as the reference category, allowing us to estimate the relative risk of participants being in either the normal or subthreshold category at follow-up compared to remaining clinically depressed.

The primary predictors included treatment group (DISCOVER vs TAU), baseline depression severity (clinical, subthreshold, or normal), and the interaction between treatment and baseline severity. Baseline severity was modelled as a categorical variable with clinical severity as the reference category, such that the main effect of treatment reflected the effect of DISCOVER among participants who were clinically depressed at baseline, and interaction terms estimated whether the effect of DISCOVER differed for those who were subthreshold or normal at baseline.

Results are presented as relative risk ratios (RRRs) with 95% confidence intervals. In addition to model coefficients, we computed adjusted marginal probabilities to aid the interpretation of predicted outcomes across groups. These marginal effects reflect the estimated probability of being in each depression category at follow-up, adjusted for baseline severity and treatment group.

#### 2.7.2 Subgroup analysis by baseline symptom severity

Subgroup analyses were conducted to examine the differential effectiveness of the DISCOVER intervention among adolescents with varying levels of depression severity at baseline. In accordance with the trial protocol, a primary subgroup analysis was planned for participants who met criteria for clinical-level depression at baseline, defined using established MFQ thresholds. An additional exploratory subgroup analysis was also conducted for those who fell within the subthreshold symptom range at baseline. For each subgroup, the primary outcome (MFQ score) and secondary outcomes were analysed using the same mixed-effect multilevel linear model described for the full sample. These models included fixed effects for baseline severity (continuous MFQ score), aggregated school-level deprivation, school size, gender, ethnicity, assessment timepoint (12 or 18 months), treatment group, and treatment-by-time interaction. These covariates were selected *a priori* on the basis of scientific rationale and were pre-specified in the trial protocol (22). Random intercepts were included for both student and school to account for clustering and repeated measurements. The adjusted mean differences between DISCOVER and TAU groups at each follow-up point were estimated alongside 95% confidence intervals and p-values. Standardised effect sizes (Cohen’s d) with 95% confidence intervals were also calculated using a pooled standard deviation. The same model structure was applied to each secondary outcome.

Cohen’s d effect sizes interpretations were informed by established conventions (28), according to which values of 0.20–0.49 are classified as small, 0.50–0.79 as moderate, and ≥0.80 as large. However, given that psychosocial interventions typically produce small or negligible effects (29, 30), and that similar interventions produce effects around 0.1 at 12 months follow-up (4), effects of 0.1 and above were considered small, and a further distinction was made between “small” (d = 0.1–0.29) and “small-to-moderate” (d = 0.3–0.49) effects. When effect sizes fall below 0.10 or span multiple categories, they are described as “negligible” (d < 0.10) or use combined terms (e.g., “negligible to small”) to accurately reflect their magnitude. This finer categorisation recognises that effect sizes approaching the moderate range may represent clinically meaningful impacts in the context of preventive mental health interventions, where even modest improvements can translate to substantial population-level benefits. This approach aligns with recommendations to contextualise effect size interpretations within the specific field of study rather than applying rigid thresholds uniformly across all research domains (31).

## 3 Results

At baseline, participants were categorised into three groups based on depression severity. In the DISCOVER group, 142 participants (32%) were classified as having clinical-level symptoms, 170 participants (38%) as subthreshold, and 131 participants (30%) as within the normal range. In the TAU group, 172 participants (38%) were classified as clinical, 157 (34%) as subthreshold, and 128 (28%) as normal.

### 3.1 Clinical depression group

**Figure 1:**
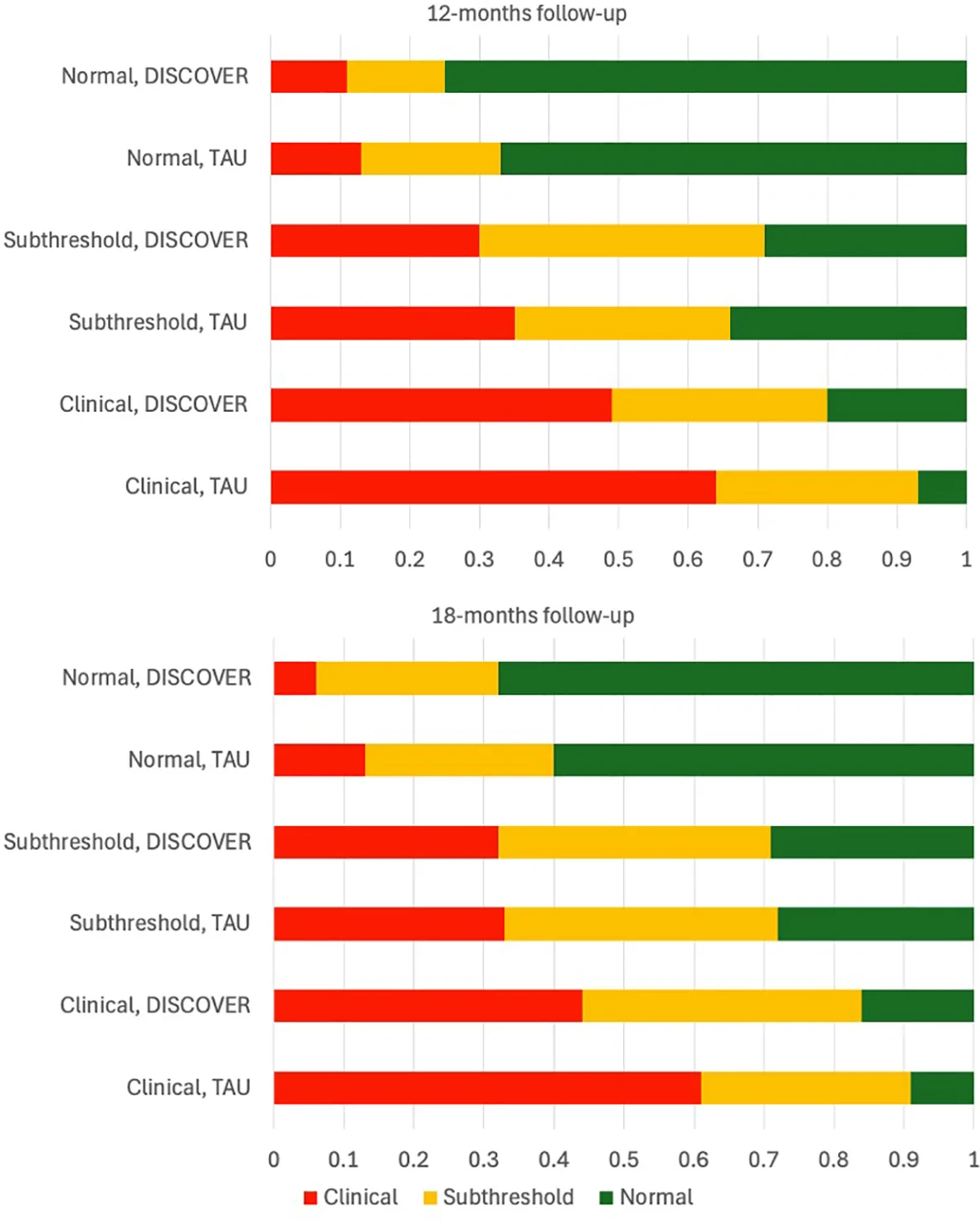
Adjusted predicted probabilities of depression severity categories at 12-month follow-up by baseline severity and treatment group. Each bar represents the probability distribution across outcome categories for adolescents in either TAU or DISCOVER, conditioned on their baseline severity classification.

DISCOVER demonstrated substantial and sustained benefits for adolescents with clinical-level depression at baseline. At 12 months, those receiving DISCOVER were three times more likely to achieve normal-range symptoms compared to TAU (RRR = 3.12, 95% CI: 1.38 to 7.05, p = 0.006). Marginal effects (**Table 1**) revealed a 20% probability of being in the normal range in DISCOVER versus only 7% in TAU, with participants also less likely to remain clinically depressed (49% vs 64%). This improved status persisted at 18 months (**Table 2**), with DISCOVER participants showing significantly higher odds of normal-range recovery (RRR = 2.63, p = 0.029; 16% vs 9% probability) and subthreshold improvement (RRR = 1.83, p = 0.044; 40% vs 30% probability), while remaining less likely to stay clinical (44% vs 61%). **Figure 1** illustrates these differential probability distributions across outcome categories by baseline severity.

**Table 1:** Adjusted marginal probabilities (with 95% confidence intervals) of depression severity outcomes at 12-month follow-up by baseline severity and treatment group.

Follow-up outcome	TAU	DISCOVER
Baseline sub-groups	Adjusted probability (95% CI)	Adjusted probability (95% CI)
**Clinical outcome score at 12m**	0.38 (0.32, 0.44)	0.30 (0.24, 0.36)
Baseline clinical	0.64 (0.55, 0.72)	0.49 (0.39, 0.59)
Baseline subthreshold	0.35 (0.27, 0.43)	0.30 (0.22, 0.38)
Baseline normal	0.13 (0.06, 0.19)	0.11 (0.04, 0.17)
**Subthreshold outcome score at 12m**	0.31 (0.26, 0.37)	0.31 (0.25, 0.37)
Baseline clinical	0.29 (0.20, 0.36)	0.31 (0.22, 0.40)
Baseline subthreshold	0.31 (0.23, 0.40)	0.41 (0.33, 0.50)
Baseline normal	0.20 (0.13, 0.28)	0.14 (0.07, 0.21)
**Normal outcome score at 12m**	0.30 (0.24, 0.36)	0.39 (0.33, 0.45)
Baseline clinical	0.07 (0.03, 0.13)	0.20 (0.12, 0.28)
Baseline subthreshold	0.34 (0.26, 0.43)	0.29 (0.21, 0.37)
Baseline normal	0.67 (0.58, 0.76)	0.76 (0.67, 0.84)

The bolded text on the left-hand side represents the classification outcome at 12 months follow-up, while the non-bolded text represents the classification outcome at baseline. Confidence intervals reflect uncertainty in predicted probabilities derived from multilevel models, accounting for clustering at the school level.

**Table 2:** Adjusted marginal probabilities (with 95% confidence intervals) of depression severity outcomes at 18-month follow-up by baseline severity and treatment group.

Group outcome	TAU	DISCOVER
Baseline sub-groups	Adjusted probability (95% CI)	Adjusted probability (95% CI)
**Clinical outcome score at 18m**	0.36 (0.30, 0.42)	0.25 (0.19, 0.31)
Baseline clinical	0.61 (0.53, 0.70)	0.44 (0.34, 0.54)
Baseline subthreshold	0.33 (0.24, 0.41)	0.32 (0.24, 0.41)
Baseline normal	0.13 (0.06, 0.16)	0.06 (0.01, 0.11)
**Subthreshold outcome score at 18m**	0.37 (0.31, 0.43)	0.40 (0.33, 0.46)
Baseline clinical	0.30 (0.22, 0.38)	0.40 (0.30, 0.50)
Baseline subthreshold	0.39 (0.30, 0.47)	0.39 (0.30, 0.48)
Baseline normal	0.27 (0.18, 0.36)	0.26 (0.17, 0.35)
**Normal outcome score at 18m**	0.27 (0.22, 0.33)	0.35 (0.29, 0.41)
Baseline clinical	0.09 (0.04, 0.13)	0.16 (0.09, 0.24)
Baseline subthreshold	0.29 (0.20, 0.37)	0.29 (0.20, 0.37)
Baseline normal	0.60 (0.50, 0.70)	0.68 (0.58, 0.77)

The bolded text on the left-hand side represents the classification outcome at 18 months follow-up, while the non-bolded text represents the classification outcome at baseline. Confidence intervals reflect uncertainty in predicted probabilities derived from multilevel models, accounting for clustering at the school level.

**Figure 2:**
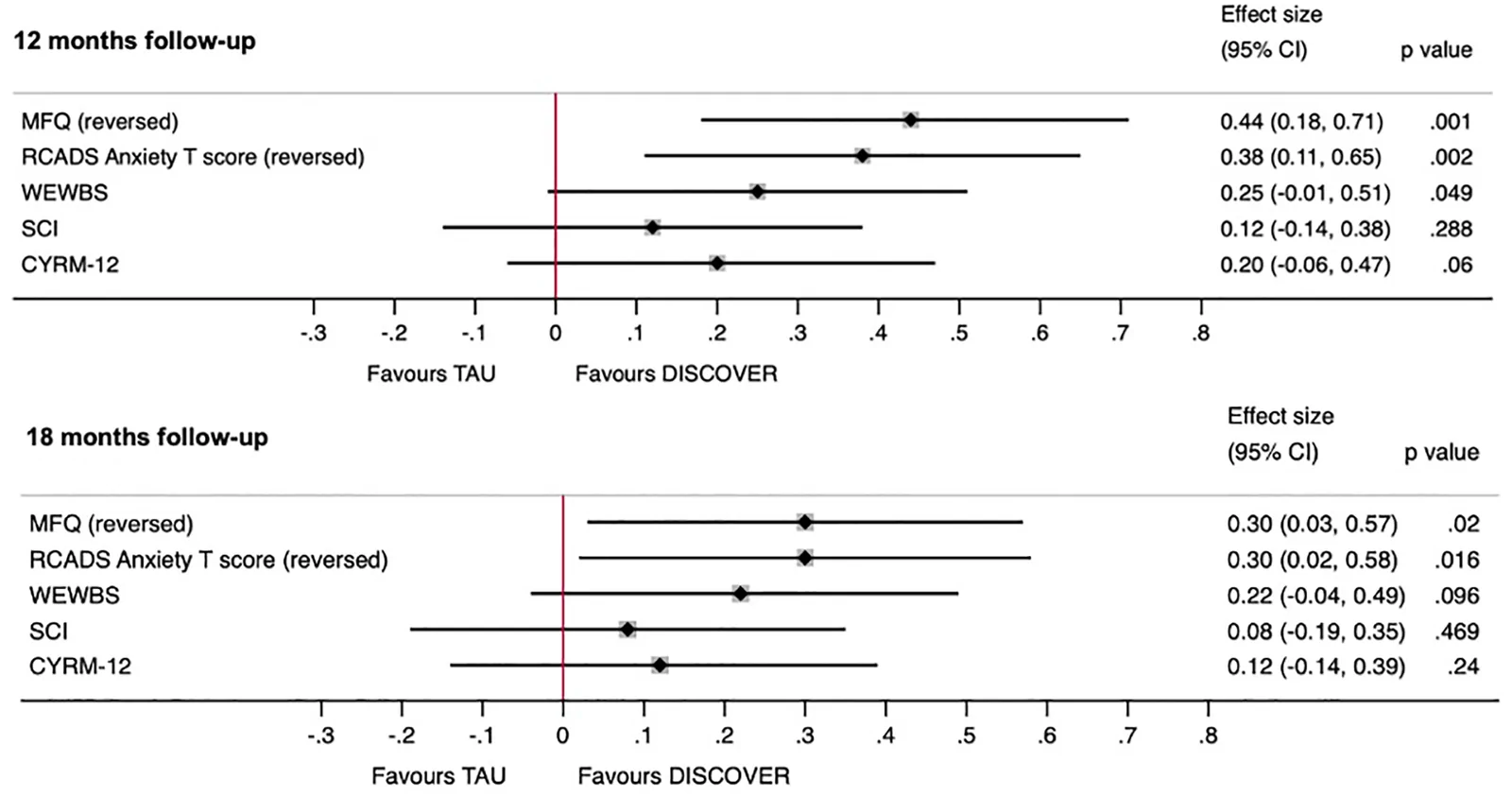
Subgroup analysis for participants with subthreshold MFQ scores at baseline. (12 months: Total N= 232, DISCOVER N=100, TAU N=132; 18 months: Total N=222, DISCOVER N=93, TAU N=129).

Continuous measures (**Figure 2**) corroborated these findings with small-to-moderate effects favouring DISCOVER at 12 months for depression (d = 0.44, 95% CI: 0.18 to 0.71, p = 0.001), anxiety (d = 0.38, 95% CI: 0.11 to 0.65, p = 0.002), and wellbeing (d = 0.25, 95% CI: -0.01 to 0.51, p = 0.049). Effects on sleep quality (d = 0.12, p = 0.288) and resilience (d = 0.20, p = 0.06) were smaller and non-significant. At 18 months, significant small-to-moderate effects persisted for depression (d = 0.30, 95% CI: 0.03 to 0.57, p = 0.02) and anxiety (d = 0.30, 95% CI: 0.02 to 0.58, p = 0.016), with small non-significant differences for wellbeing (d = 0.22, p = 0.096) and resilience (d = 0.12, p = 0.24), and negligible differences for sleep quality (d = 0.08, p = 0.469).

**Figure 3:**
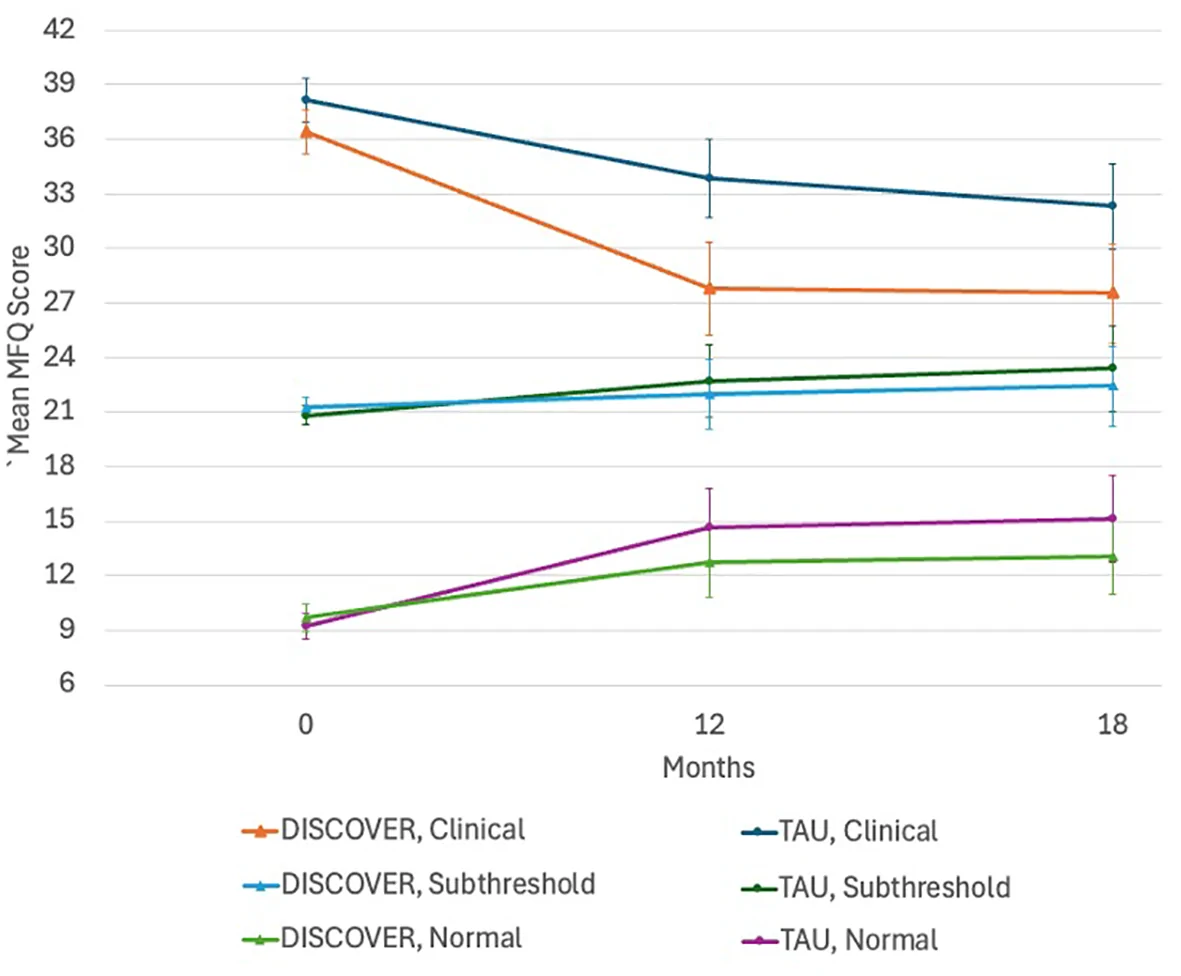
Temporal plot showing the mean MFQ scores for participants with clinical, subthreshold and normal depression symptom severity at baseline. At 12 months, DISCOVER showed a greater reduction in depressive symptoms than TAU among participants with clinical baseline symptoms (d = 0.44, 95% CI: 0.18 to 0.71, p = 0.001), with effects remaining significant at 18 months (d = 0.30, 95% CI: 0.03 to 0.57, p = 0.02). No statistically significant differences were observed for subthreshold or normal baseline groups. (DISCOVER Clinical: 12m N=100, 18m N=93, DISCOVER Subthreshold: 12m N=121, 18m N=115, DISCOVER Normal: 12m N=94, 18m N=93, TAU Clinical: 12m N=132; 18m TAU N=129, TAU Subthreshold: 12m N=120; 18m N=119, TAU Normal: 12m N=103; 18m N=100).Temporal plot showing the mean wellbeing (WEMWBS) scores for participants with clinical, subthreshold and normal depression symptom severity at baseline. At 12 months, a small difference favouring DISCOVER was observed among participants with clinical baseline symptoms (d = 0.25, 95% CI: -0.01 to 0.51, p = 0.049), although this did not remain statistically significant at 18 months (d = 0.22, p = 0.096). No statistically significant differences were observed for subthreshold or normal baseline groups. (DISCOVER Clinical: 12m N = 100, 18m N = 93, DISCOVER Subthreshold: 12m N = 121, 18m N = 115, DISCOVER Normal: 12m N = 94, 18m N = 93, TAU Clinical: 12m N = 132; 18m TAU N = 129, TAU Subthreshold: 12m N = 120; 18m N = 119, TAU Normal: 12m N = 103; 18m N = 100).

**Figure 4:**
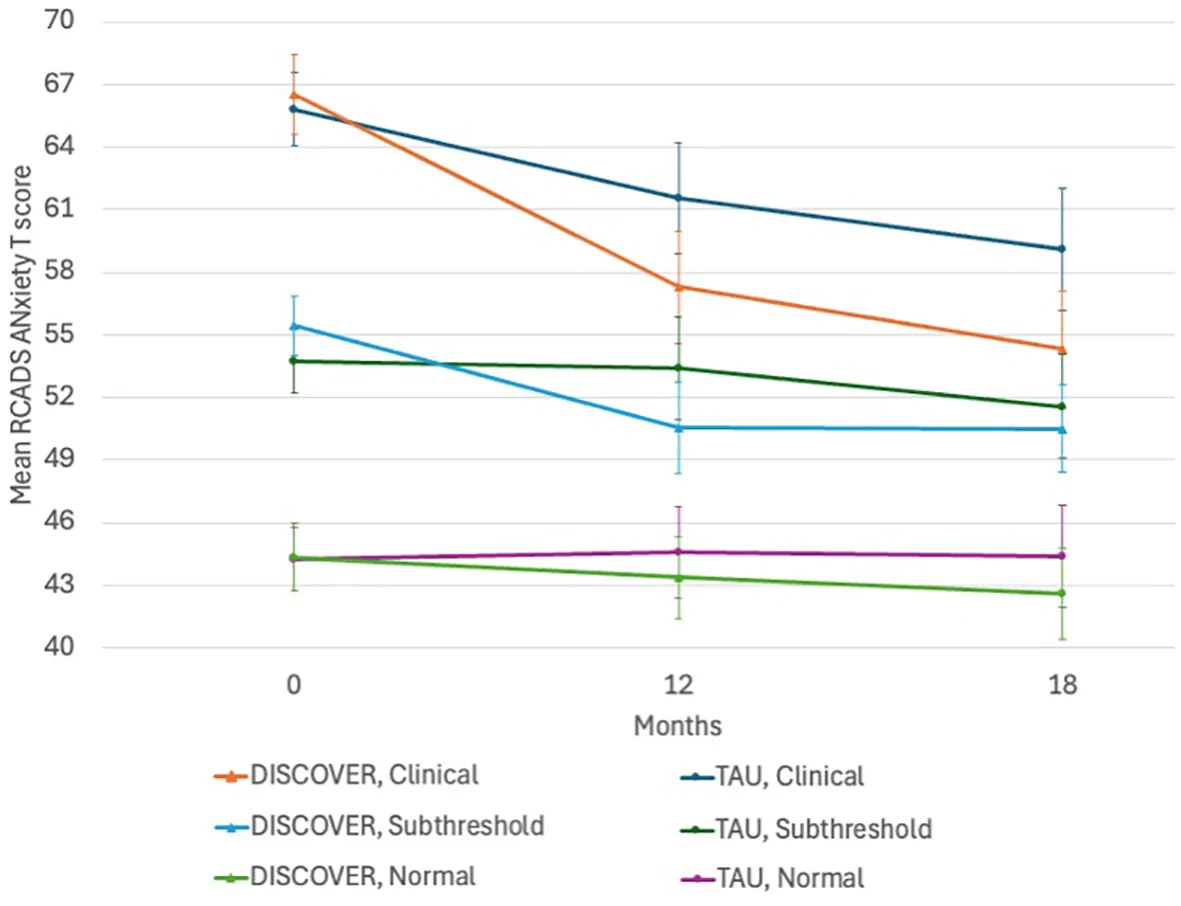
Temporal plot showing the mean RCADS Anxiety T scores for participants with clinical, subthreshold and normal depression symptom severity at baseline. At 12 months, DISCOVER showed greater reductions in anxiety symptoms compared to TAU among participants with clinical (d = 0.38, 95% CI: 0.11 to 0.65, p = 0.002) and subthreshold baseline symptoms (d = 0.34, 95% CI: 0.08 to 0.60, p = 0.004). At 18 months, effects remained significant for the clinical group (d = 0.30, 95% CI: 0.02 to 0.58, p = 0.016), while differences for subthreshold participants were no longer statistically significant. (DISCOVER Clinical: 12m N=100, 18m N=93, DISCOVER Subthreshold: 12m N=121, 18m N=115, DISCOVER Normal: 12m N=94, 18m N=93, TAU Clinical: 12m N=132; 18m TAU N=129, TAU Subthreshold: 12m N=120; 18m N=119, TAU Normal: 12m N=103; 18m N=100).

**Figure 5:**
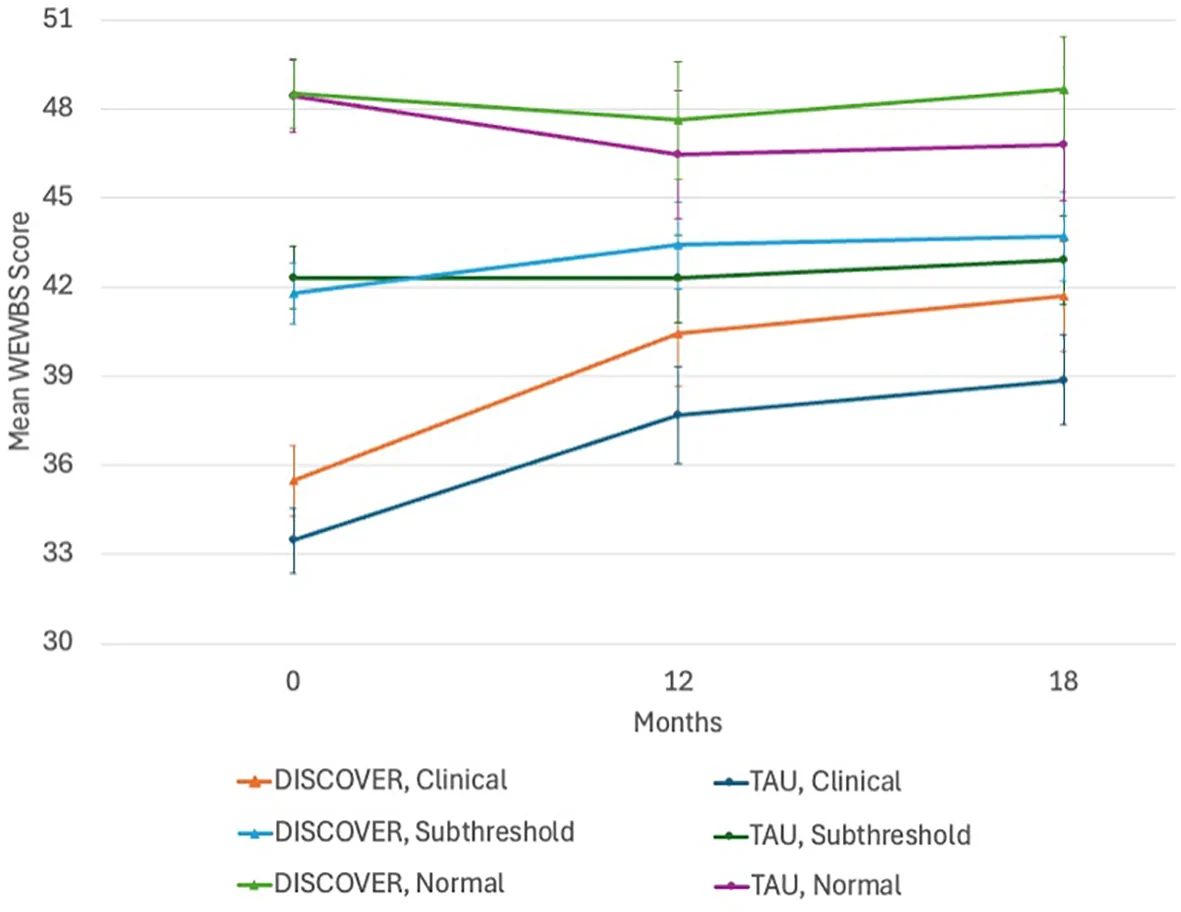
Temporal plot showing the mean wellbeing (WEMWBS) scores for participants with clinical, subthreshold and normal depression symptom severity at baseline. At 12 months, a small difference favouring DISCOVER was observed among participants with clinical baseline symptoms (d = 0.25, 95% CI: -0.01 to 0.51, p = 0.049), although this did not remain statistically significant at 18 months (d = 0.22, p = 0.096). No statistically significant differences were observed for subthreshold or normal baseline groups. (DISCOVER Clinical: 12m N=100, 18m N=93, DISCOVER Subthreshold: 12m N=121, 18m N=115, DISCOVER Normal: 12m N=94, 18m N=93, TAU Clinical: 12m N=132; 18m TAU N=129, TAU Subthreshold: 12m N=120; 18m N=119, TAU Normal: 12m N=103; 18m N=100).

**Figure 6:**
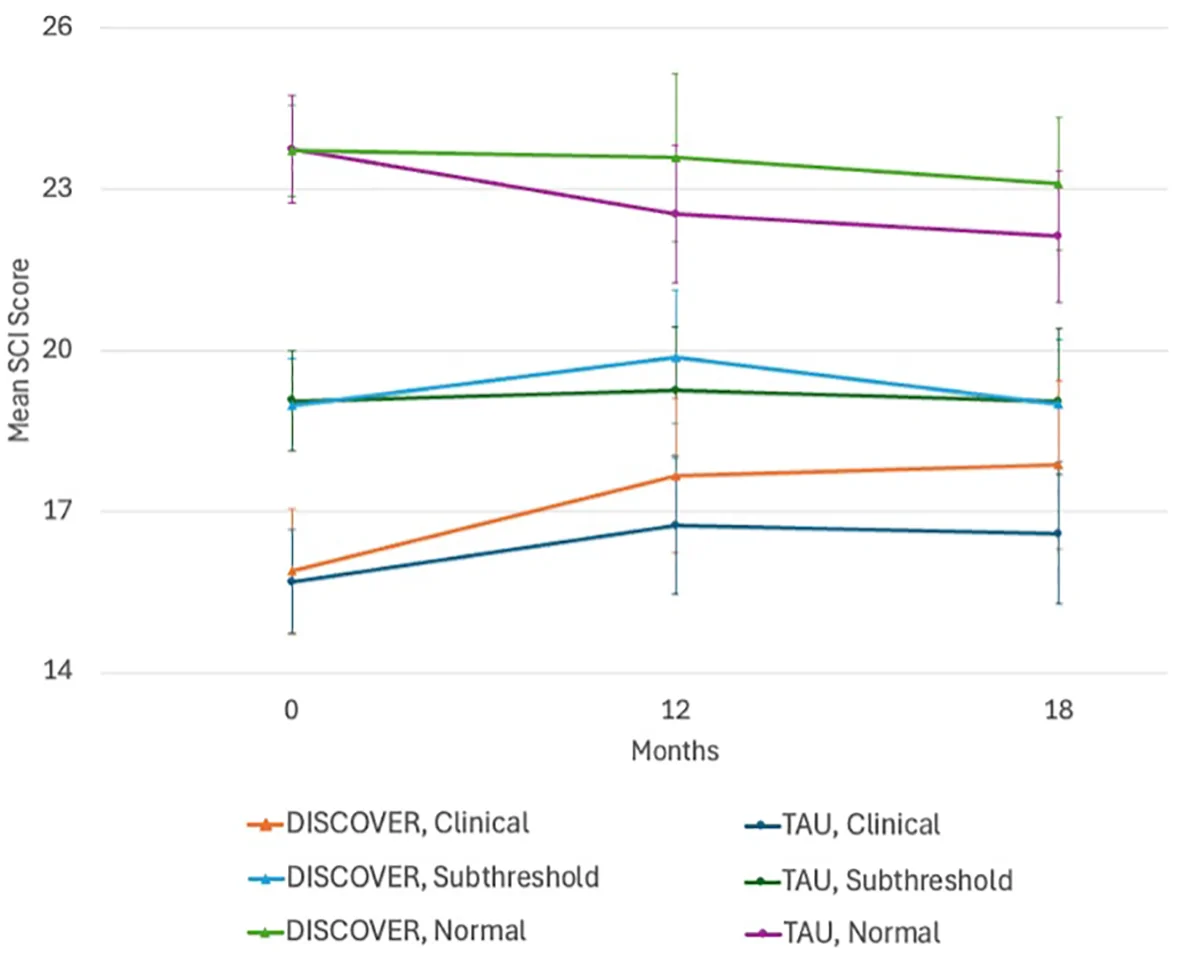
Temporal plot showing the sleep quality (SCI) scores for participants with clinical, subthreshold and normal depression symptom severity at baseline. No statistically significant differences between DISCOVER and TAU were observed for sleep quality at either 12 months (clinical group: d = 0.12, p = 0.288) or 18 months (d = 0.08, p = 0.469), with similarly negligible effects across subthreshold and normal baseline groups. (DISCOVER Clinical: 12m N = 100, 18m N = 93, DISCOVER Subthreshold: 12m N = 121, 18m N = 115, DISCOVER Normal: 12m N = 94, 18m N = 93, TAU Clinical: 12m N = 132; 18m TAU N = 129, TAU Subthreshold: 12m N = 120; 18m N = 119, TAU Normal: 12m N = 103; 18m N = 100).

**Figure 7:**
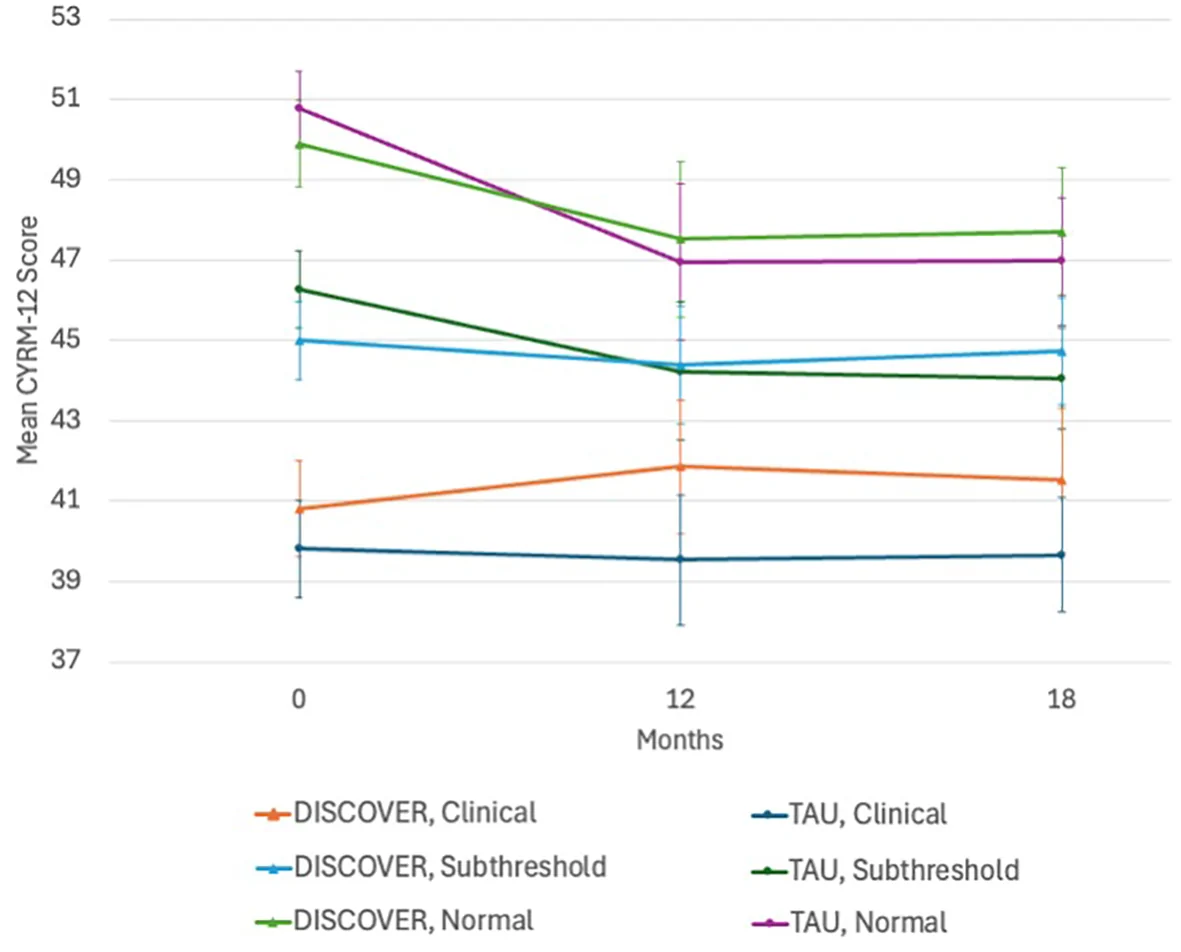
Temporal plot showing the mean resilience (CYRM-12) scores for participants with clinical, subthreshold and normal depression symptom severity at baseline. Small, non-significant differences favouring DISCOVER were observed for resilience among participants with clinical baseline symptoms at 12 months (d = 0.20, p = 0.06) and 18 months (d = 0.12, p = 0.24), with no statistically significant differences across subthreshold or normal baseline groups. (DISCOVER Clinical: 12m N = 100, 18m N = 93, DISCOVER Subthreshold: 12m N = 121, 18m N = 115, DISCOVER Normal: 12m N = 94, 18m N = 93, TAU Clinical: 12m N = 132; 18m TAU N = 129, TAU Subthreshold: 12m N = 120; 18m N = 119, TAU Normal: 12m N = 103; 18m N = 100).

Symptom trajectories (**Figures 3**–**7**) revealed that DISCOVER participants with clinical-level depressive symptoms exhibited a steeper decline from baseline to 12 months, with scores stabilising at 18 months, while TAU participants experienced a more modest and continuous decline. This pattern was evident for both depression (**Figure 3**) and anxiety symptoms (**Figure 4**), where DISCOVER participants showed larger and sustained reductions.

### 3.2 Subthreshold depression group

**Figure 8:**
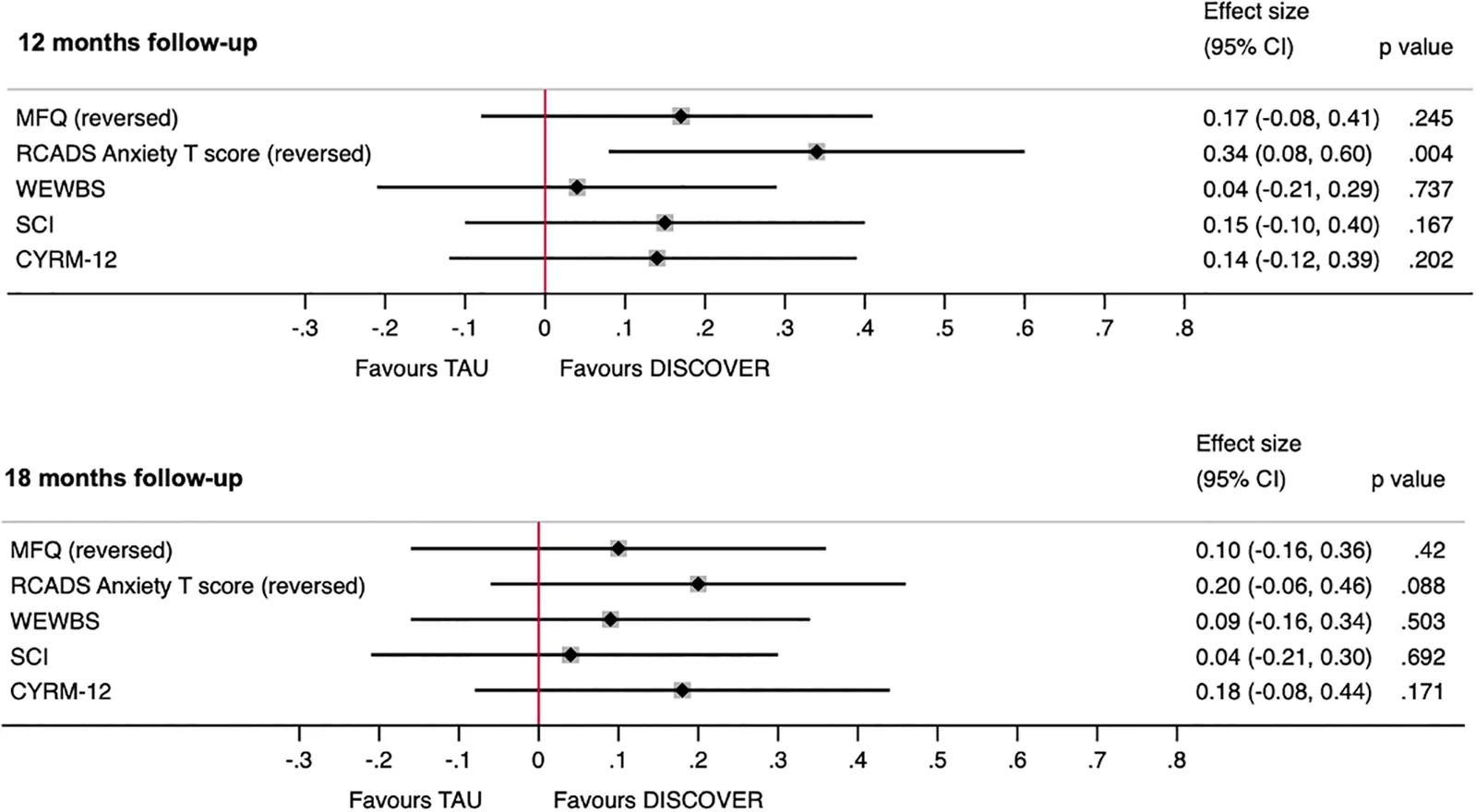
Subgroup analysis for participants with subthreshold MFQ scores at baseline. (12 months: Total N= 221, DISCOVER N=121, TAU N=120; 18 months: Total N=234, DISCOVER N=115, TAU N=119).

For participants with subthreshold depression symptoms at baseline, DISCOVER demonstrated a therapeutic effect specific to anxiety rather than depression. At 12 months, a small-to-moderate significant effect favouring DISCOVER was found for anxiety (d = 0.34, 95% CI: 0.08 to 0.60, p = 0.004; **Figure 8**), indicating the intervention addressed anxious symptomatology in this at-risk group.

However, the intervention had significantly weaker effects on categorical depression outcomes compared to the clinical group (interaction RRR = 0.32, 95% CI: 0.11 to 0.9, p = 0.030). Marginal effects (**Table 1**) showed that DISCOVER increased the probability of remaining subthreshold (41% vs 31%) but did not increase the probability of achieving normal range (29% vs 34%). Negligible to small differences in depression (d = 0.17, 95% CI: -0.08 to 0.41, p = 0.245), wellbeing (d = 0.04, 95% CI: -0.21 to 0.29, p = 0.737), sleep quality (d = 0.15, 95% CI: -0.10 to 0.40, p = 0.167), and resilience (d = 0.14, 95% CI: -0.12 to 0.39, p = 0.202) were not statistically significant.

By 18 months (**Table 2**), the relative risk of being in the normal range did not significantly differ between DISCOVER and TAU (interaction RRR = 0.39, 95% CI: 0.13 to 1.15, p = 0.089). Outcomes were virtually identical, with 29% achieving normal range and 39% remaining subthreshold in both arms. All continuous outcomes showed negligible to small non-significant differences: depression (d = 0.10, 95% CI: -0.16 to 0.36, p = 0.42), anxiety (d = 0.20, 95% CI: -0.06 to 0.46, p = 0.088), wellbeing (d = 0.09, 95% CI: -0.16 to 0.34, p = 0.503), sleep (d = 0.04, 95% CI: -0.21 to 0.30, p = 0.692), and resilience (d = 0.18, 95% CI: -0.08 to 0.44, p = 0.171; **Figure 8**).

Temporal trajectories revealed that participants with subthreshold symptoms at baseline displayed relatively flat patterns across all outcomes, with minimal change over time in either group (**Figures 3**–**7**).

### 3.3 Minimal symptoms group

**Figure 9:**
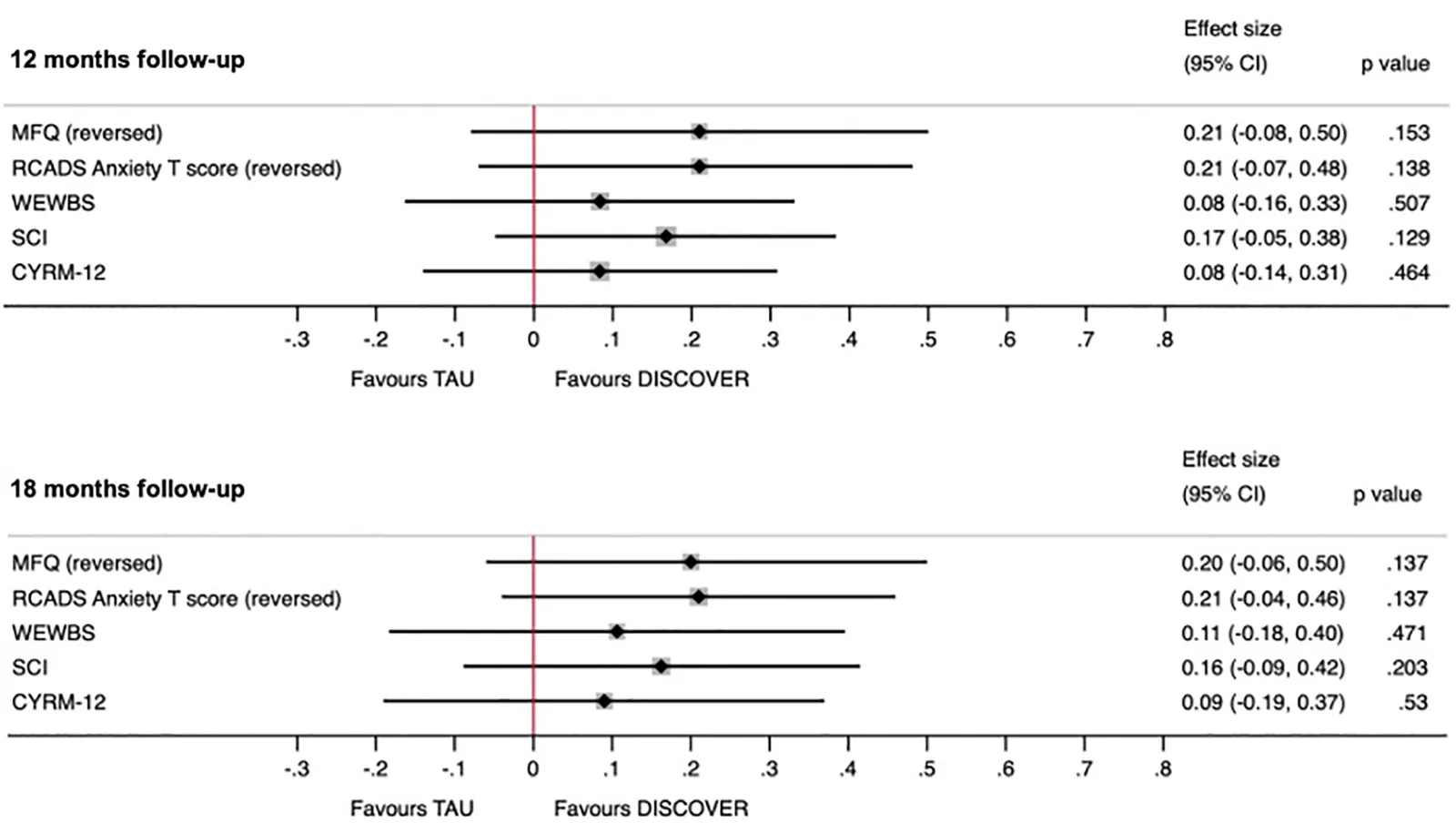
Temporal plot showing the mean RCADS Anxiety T scores for participants with clinical, subthreshold and normal depression symptom severity at baseline. At 12 months, DISCOVER showed greater reductions in anxiety symptoms compared to TAU among participants with clinical (d = 0.38, 95% CI: 0.11 to 0.65, p = 0.002) and subthreshold baseline symptoms (d = 0.34, 95% CI: 0.08 to 0.60, p = 0.004). At 18 months, effects remained significant for the clinical group (d = 0.30, 95% CI: 0.02 to 0.58, p = 0.016), while differences for subthreshold participants were no longer statistically significant. (DISCOVER Clinical: 12m N = 100, 18m N = 93, DISCOVER Subthreshold: 12m N = 121, 18m N = 115, DISCOVER Normal: 12m N = 94, 18m N = 93, TAU Clinical: 12m N = 132; 18m TAU N = 129, TAU Subthreshold: 12m N = 120; 18m N = 119, TAU Normal: 12m N = 103; 18m N = 100).

For participants who entered the trial with minimal depression symptoms at baseline, DISCOVER showed no statistically significant effects on any outcome at either timepoint (**Figure 9**). No statistically significant intervention effects were observed for adolescents with minimal baseline symptoms. Although the direction of estimates suggested higher probability of maintaining normal symptoms in the DISCOVER group compared to TAU at 12 months, confidence intervals overlapped (DISCOVER: 76%, 95% CI: 67% to 84% vs TAU: 67%, 95% CI: 58% to 76%) (**Table 1**). At 18 months (**Table 2**), the probability of maintaining normal-range symptoms remained higher in DISCOVER (68%) compared to TAU (60%), and the probability of deteriorating to the clinical range was lower in DISCOVER (6%) compared to TAU (13%).

Effect sizes for depression and anxiety were small but non-significant at both 12 months (d = 0.21, 95% CI: -0.08 to 0.50, p = 0.153 for depression; d = 0.21, 95% CI: -0.07 to 0.48, p = 0.138 for anxiety) and 18 months (d = 0.20, 95% CI: -0.06 to 0.50, p = 0.137 for depression; d = 0.21, 95% CI: -0.04 to 0.46, p = 0.137 for anxiety). Effects on wellbeing, sleep quality, and resilience were negligible to small and non-significant at both 12 months (wellbeing: d = 0.08, 95% CI: -0.16 to 0.33, p = 0.507; sleep: d = 0.17, 95% CI: -0.05 to 0.38, p = 0.129; resilience: d = 0.08, 95% CI: -0.14 to 0.31, p = 0.464) and 18 months (wellbeing: d = 0.11, 95% CI: -0.18 to 0.40, p = 0.471; sleep: d = 0.16, 95% CI: -0.09 to 0.42, p = 0.203; resilience: d = 0.09, 95% CI: -0.19 to 0.37, p = 0.530).

Symptom trajectories showed that outcomes remained stable with little variation across the follow-up period; depression and anxiety scores remained low, while wellbeing (**Figure 5**), sleep quality (**Figure 6**), and resilience scores (**Figure 7**) stayed relatively high in both arms. However, participants in the TAU group appeared to worsen slightly more than DISCOVER participants, although this could reflect regression towards the mean.

## 4 Discussion

Most school-based intervention trials report no effects, potentially masking important variation in how adolescents with different symptom severity respond to treatment. This study addressed this gap by examining whether DISCOVER’s effects at 12- and 18-month follow-ups differed by baseline depression severity. Three key patterns emerged: substantial and sustained benefits for adolescents with clinical-level depression, specific anxiety reduction for those with subthreshold symptoms, and potential protective effects for those with minimal symptoms at baseline.

### 4.1 Significant effects for clinical-level depression symptoms

DISCOVER was effective for adolescents who had clinical-level symptoms of depression. These participants were significantly more likely to transition to lower severity categories (subthreshold or normal) at both 12- and 18-month follow-ups compared to those receiving treatment-as-usual, with over three times the odds of achieving full recovery (RRR = 3.12, p = 0.006). The intervention also produced sustained improvements in depression and anxiety symptoms in this group, with small-to-moderate effect sizes maintained across timepoints.

Comparing with the 6-month clinical subgroup analysis (32), the long-term follow-up confirms that DISCOVER produced enduring benefits for adolescents with clinical-level depression. The moderate effect on depressive symptoms observed at 6 months (Cohen’s d = 0.52) diminished slightly but remained significant at 12 months (d = 0.44) and 18 months (d = 0.30). For anxiety symptoms, the effect observed at 6 months (d = 0.35) was maintained consistently at both 12 months (d = 0.38) and 18 months (d = 0.30), suggesting a particularly sustained intervention impact. However, improvements in wellbeing, resilience, and sleep quality observed at 6 months (d = 0.48, 0.27, and 0.23, respectively) were not maintained over the long term. This finding suggests that these domains may require additional support over the long term, possibly because the young people found strategies taught for managing depression and anxiety more relevant for subsequent difficulties or easier to maintain through consistent practice, while strategies like sleep hygiene need to be adapted for changes in environments and circumstances which occur in early young adulthood.

These sustained effects are particularly encouraging given evidence that intervention effects for high-risk groups often diminish over time (4). A meta-analysis of targeted school-based interventions found that while these programmes often yielded small-to-moderate reductions in depressive symptoms immediately post-intervention (SMD = -0.34), effects attenuated to non-significance at medium- and long-term follow-up (33). Similarly, recent large-scale trials of universal school-based interventions—including mindfulness training delivered by teachers (16), classroom-based CBT (34), and digital interventions (35)—found no differences or even worse outcomes at 12-month follow-up for students with clinical-level depression. The finding that DISCOVER participants with clinical-level depression had significantly higher odds of full recovery (RRR = 3.12) also contrasts favourably with the more modest effects on recovery observed in meta-analyses of indicated CBT interventions (RR = 0.64; 36).

### 4.2 Encouraging but specific benefits for subthreshold depression

For adolescents with subthreshold symptoms at baseline, DISCOVER demonstrated a more specific pattern of effects. The finding of a significant small-to-moderate effect on anxiety symptoms at 12 months (d = 0.34, p = 0.004) is encouraging and suggests that the intervention successfully engaged this at-risk group in reducing anxious distress. This anxiety-specific benefit may reflect several factors. First, adolescents in the subthreshold group may have presented with anxiety-dominant or mixed symptom profiles. The presentation of anxiety itself could explain why anxiety outcomes were more responsive to the intervention than depressive symptoms: anxiety symptoms tend to fluctuate in response to changing stressors, providing more frequent opportunities over time for adolescents to apply and reinforce the coping strategies introduced in the intervention. In contrast, subthreshold depressive symptoms, particularly those characterised by anhedonia or low motivation, may offer fewer opportunities for active skill use and may require more sustained or intensive input to produce meaningful change. Second, the cognitive and behavioural strategies taught in DISCOVER, such as cognitive restructuring, exposure techniques, and worry management, may align particularly well with the presenting concerns of adolescents experiencing primarily anxious symptomatology alongside subthreshold low mood. For example, adolescents consistently identified personalised goal-setting components as among the most meaningful and actively used elements of the intervention (37). These components were often described as directly applicable to managing day-to-day stressors and anxiety-related difficulties, such as worry, avoidance, and feeling overwhelmed, rather than more persistent or diffuse depressive symptoms such as low mood or anhedonia. Furthermore, engagement with intervention content may be dynamic rather than static, with adolescents revisiting and applying different skills as their needs evolve over time, potentially favouring the continued use of anxiety-focused strategies. This highlights the importance of moving beyond one-time engagement metrics to capture how skills are used and reapplied over time, as well as the need for longitudinal assessment of skill uptake to better understand mechanisms of sustained change.

However, this anxiety benefit did not extend to broader improvements in depression, wellbeing, resilience, or sleep quality, and effects were no longer significant by 18 months. This pattern of limited broader improvement is consistent with a recent meta-analysis which found significant short-term reductions in depressive symptoms from psychological interventions for adolescents with subthreshold depression, but no significant effects at 6–18 months follow-up in preventing clinical depression (38). Importantly, as child and adolescent psychiatrists have noted, the lack of statistical significance in these findings might be related to limited statistical power and wide confidence intervals, suggesting that larger sample sizes could reveal clearer preventive effects (39). This limitation applies equally to the BESST trial, which was not adequately powered to detect smaller preventive effects over 18 months in the subthreshold subgroup specifically. Therefore, these findings should be interpreted cautiously and cannot definitively distinguish intervention effects from natural symptom variation.

The limited broader effects for those with subthreshold symptoms may also reflect the nature of subthreshold depression itself, with a restricted symptom range, which can limit observable pre–post change (38). Adolescents in this group may experience variable levels of motivation, readiness for change, and symptom awareness that affect engagement and impact (40). Their distress levels may not yet be sufficiently high to create strong motivation to engage fully with all intervention content, or certain intervention strategies may feel less personally relevant (40, 41). Additionally, subthreshold symptoms may be more heterogeneous than clinical depression, with diverse underlying causes requiring different intervention approaches (42). Some adolescents with subthreshold symptoms may primarily need support for anxiety (which DISCOVER successfully provided), while others may require different support for emerging depression, highlighting the need for personalised interventions (43). Stratifying adolescents based primary presenting concern (anxiety-predominant vs. depression-predominant vs. mixed) and delivering different intervention components or follow-up support catered to their presenting needs should be a priority for future research.

One possible explanation is that anxiety symptoms, which tend to fluctuate in response to changing stressors, may provide more frequent opportunities for adolescents to apply and reinforce the skills introduced in the intervention over time. In contrast, subthreshold depressive symptoms, particularly those characterised by anhedonia or low motivation, may offer fewer opportunities for active skill use and may require more sustained intervention to produce meaningful change. Additionally, adolescents may engage with intervention components dynamically, revisiting and applying different strategies as their needs evolve, which may favour the continued use of anxiety-focused techniques over time.

### 4.3 Potential protective effects for minimal symptoms

Participants in the normal range at baseline showed no statistically significant intervention effects but demonstrated descriptive patterns suggesting potential protective benefits. DISCOVER participants maintained higher rates of normal-range symptoms and lower rates of deterioration to clinical depression compared to TAU, though these differences did not reach statistical significance. This pattern suggests that brief school-based interventions may offer some protective value even for adolescents without current symptoms, although the absence of statistical significance and limited power preclude firm conclusions.

### 4.4 Strengths and limitations

The rigorous methodological design within a cluster randomised controlled trial provides robust evidence on the effectiveness of the DISCOVER intervention across varying levels of depression severity (44). The use of long-term follow-up (12- and 18-months post-intervention) significantly contributes to our understanding of sustained intervention effects, addressing a gap in existing literature where most school-based mental health interventions are evaluated only in the short term.

Several limitations should be acknowledged. First, the use of predefined MFQ cut-offs to categorise baseline severity introduces some uncertainty, as reported MFQ cut-off values for clinical depression range from ≥22 to ≥29 (45), and there is no universally accepted threshold. However, the chosen thresholds were selected from robust studies that closely mirrored the characteristics of the current trial population, including help-seeking UK adolescents (26, 27), and have recently been used in another other large UK trial (46), enhancing confidence in their validity.

Second, the subgroup analysis approach itself faces inherent limitations related to statistical power. The smaller size of subgroup populations means they were not powered to detect differences, particularly for the subthreshold and normal groups. Findings should therefore be interpreted with caution, as non-significant results may reflect insufficient power rather than true lack of efficacy. This is particularly relevant for interpreting the limited broader effects observed in adolescents with subthreshold depression symptoms.

Third, variation in symptom trajectories in the treatment-as-usual (TAU) group complicates interpretation. Participants with clinical-level depression in TAU showed improvements over time, though less pronounced than those receiving DISCOVER, possibly reflecting natural symptom fluctuation, regression to the mean, or beneficial effects of standard school support and maturation. However, TAU trajectories were flatter for subthreshold participants and showed slight worsening for those in the normal range at baseline. This heterogeneity in control group outcomes may have reduced detectable differences between groups, particularly for participants with subthreshold symptoms.

Fourth, the study was limited by the lack of participant blinding and reliance on self-report measures. Participants were aware of their treatment allocation, which may have introduced expectancy effects or influenced symptom reporting.

Finally, an important limitation relates to the reliance on self-report questionnaire measures to assess depressive symptoms. While the Mood and Feelings Questionnaire (MFQ) is a well-validated screening tool, changes in questionnaire scores cannot be equated with clinical remission or recovery, since “clinical” group represents elevated MFQ scores, not confirmed diagnoses.

ad. In the absence of structured diagnostic interviews (e.g., the Kiddie Schedule for Affective Disorders and Schizophrenia; K-SADS), it is not possible to determine whether participants who moved below clinical thresholds experienced true remission, nor whether new or recurrent depressive episodes occurred during the follow-up period. However, based on prior validation studies of the MFQ, about 78% of the “clinical” group likely met DSM-5 criteria for Major Depressive Disorder (26). This distinction is particularly important when interpreting longer-term outcomes and potential preventive effects, as apparent improvements or stability in symptom scores may reflect measurement limitations, regression to the mean, or natural fluctuations rather than sustained recovery or prevention of disorder onset. As such, findings should be interpreted as reflecting changes in symptom severity rather than definitive clinical outcomes.

### 4.5 Implications

The results of this study offer support for self-referral, school-based CBT interventions such as DISCOVER, particularly for adolescents experiencing clinical-level depression who are not yet in contact with specialist mental health services. These outcomes are important given the well-documented difficulties in engaging this high-risk group through traditional service routes, and the limited long-term effectiveness of many targeted interventions for adolescent depression (33).

From a service design perspective, the results suggest that self-referral models can serve as an effective alternative to both universal and targeted screening-based approaches. Compared to universal interventions often delivered by teachers, which students with elevated symptoms may perceive as too generic or “watered down,” self-referral models offering DISCOVER delivered by clinicians may offer a better balance between accessibility and targeted support (47). Furthermore, unlike screening-based targeted interventions, which may miss some young people or carry stigma, self-referral enables young people to opt in based on personal recognition of distress (47). This approach was successful in reaching adolescents with clinically significant symptoms, many of whom had not previously accessed specialist services (48), and may thus represent an effective mechanism for early intervention in educational settings.

Furthermore, DISCOVER achieved greater quality-adjusted life-years (QALYs) at lower total costs per student, representing extremely strong evidence of economic value (44). These findings highlight the economic importance of addressing mental health issues during adolescence, as even modest improvements in psychological wellbeing can translate into significant productivity gains as young people transition into the workforce (49).

There have been few studies on interventions specifically targeting sub-threshold depression (50–52). The encouraging but more limited effects for adolescents with subthreshold symptoms—particularly the specific anxiety benefits—raise important questions about how to optimise outcomes for this group. Stepped-care approaches, in which individuals begin with low-intensity interventions and receive more intensive support only if symptoms persist, have been proposed as efficient ways to match treatment intensity to need and improve access to care (53, 54). Evidence also suggests that stepped collaborative care models can be feasible and acceptable for adolescents with depression (55). Alternatively, offering modular content that allows adolescents to select strategies based on their individual needs and preferences (56, 57) may enhance relevance and increase engagement (58). Given that anxiety reduction was achieved, future iterations could explore how to build on this success to promote broader wellbeing gains, perhaps by extending intervention duration for this group or providing booster sessions to maintain and expand initial benefits. Future research could also explicitly test adaptive models of care, including personalised follow-up pathways that build on initial intervention exposure and respond to evolving symptom profiles over time.

Future research should also explore how adolescents with subthreshold symptoms perceive such interventions and identify which strategies can increase relevance and engagement (59). Qualitative investigation into why anxiety symptoms responded while depression and wellbeing did not could inform intervention refinement. Understanding the heterogeneity within the subthreshold group—for example, distinguishing those primarily experiencing anxiety from those experiencing emerging depression (57, 60) or matching interventions to different risk profiles (43) —may enable more targeted support.

### 4.6 Conclusion

These findings support the utility of a self-referral school-based intervention model for adolescents experiencing clinical-level symptoms who may not yet be engaged with specialist services. The observed sustained effects at 12- and 18-month follow-up suggest that DISCOVER can help mitigate the progression of clinical-level depression and anxiety when delivered in accessible, non-stigmatising school environments. The encouraging finding of specific anxiety reduction for adolescents with subthreshold symptoms indicates that the intervention has value for this at-risk group.

## Data Availability

The raw data supporting the conclusions of this article will be made available by the authors, without undue reservation.
