# Supplementary material for: Do all adolescents benefit equally from school-based mental health interventions? A subgroup analysis by depression severity at 12 and 18 months follow-up
Source: Front Psychiatry. 2026 Aug 10;17:1826587. doi: 10.3389/fpsyt.2026.1826587 (PMC13501227; doi:10.3389/fpsyt.2026.1826587)
Supplement: Supplementary file 1 [file Table1.docx]

Supplementary Table 1. Treatment-as-usual (TAU) provision across participating schools

| **Provision** | **DISCOVER Arm (%)** | **TAU Arm (%)** |
| --- | --- | --- |
| **Universal school/college provision** |  |  |
| Promotion of mental health and wellbeing integrated into the school day | 80 | 83 |
| Provision of information and signposting to online/external support | 52 | 45 |
| A worry box/drop-in for advice and signposting | 76 | 66 |
| Activities to reduce stigma of mental health | 84 | 93 |
| An ethos/environment promoting mutual care and respect for diversity | 64 | 52 |
| Opportunities for pupils to be involved in wellbeing decisions | 52 | 48 |
| Peer mentoring/support | 88 | 86 |
| Learning about mental health in curriculum (e.g., PSHE lessons) | 84 | 72 |
| Sessions on specific issues (e.g., body image, eating disorders, self-harm) | 80 | 72 |
| Skills sessions (e.g., coping, problem-solving, mindfulness) | 80 | 83 |
| **Targeted support for individual pupils** |  |  |
| Counselling | 32 | 17 |
| Cognitive Behavioural Therapy (CBT) | 68 | 45 |
| One-to-one support for specific issues | 72 | 69 |
| Skills sessions (targeted) | 36 | 24 |
| Themed support groups (e.g., anxiety, eating disorders) | 44 | 10 |
| Anger management groups | 40 | 24 |
| Peer-led support | 44 | 34 |
| Nurture groups | 40 | 48 |
| Support programmes for specific vulnerable groups | 92 | 90 |
